# Supplementary material for: SIRT7 suppresses energy expenditure and thermogenesis by regulating brown adipose tissue functions in mice
Source: Nat Commun. 2022 Dec 12;13:7439. doi: 10.1038/s41467-022-35219-z (PMC9744749; doi:10.1038/s41467-022-35219-z)
Supplement: Supplementary file 1 — Supplementary Information [file 41467_2022_35219_MOESM1_ESM.pdf]

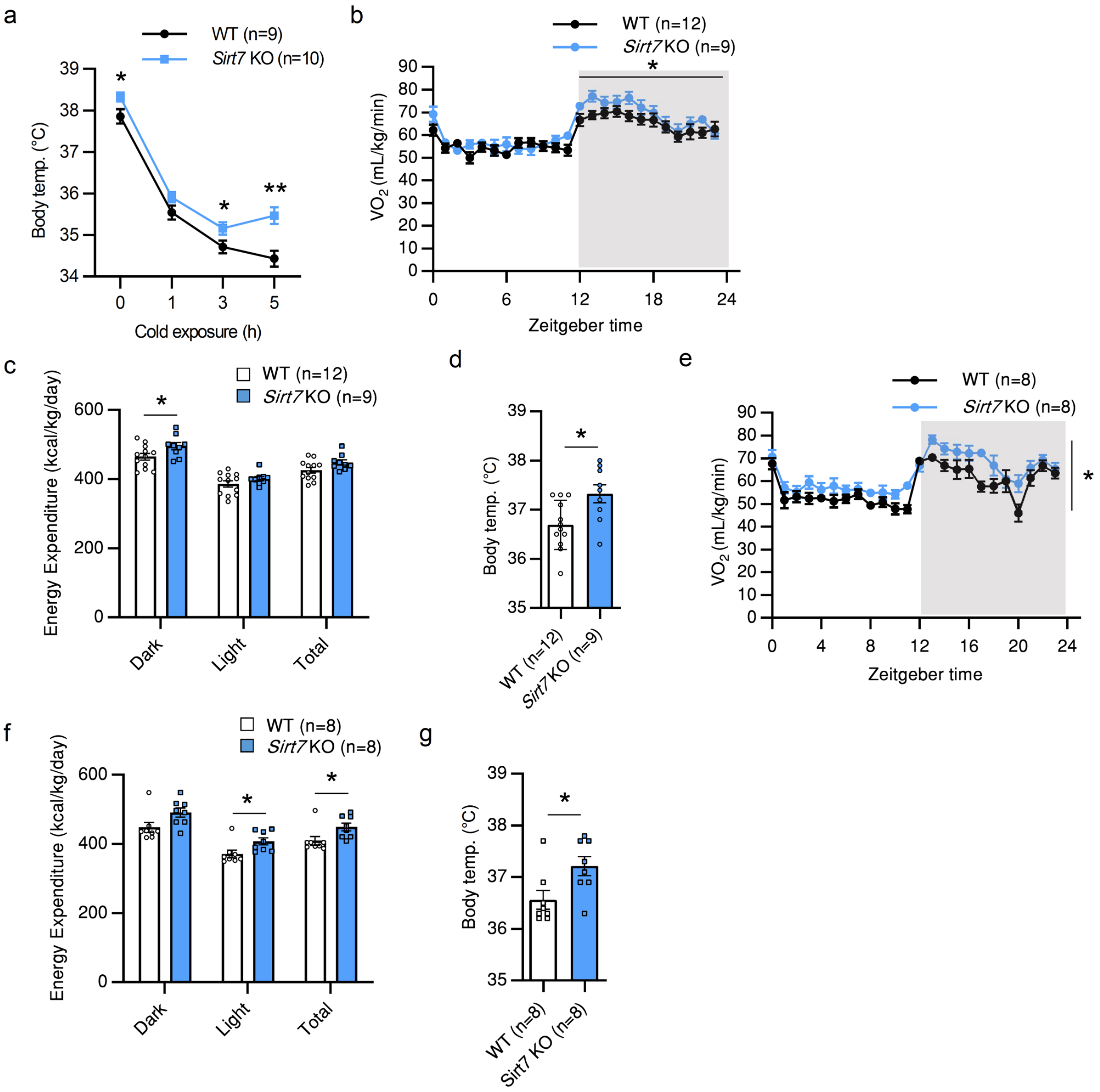

a
WT (n=9)
Sirt7 KO (n=10)
Body temp. (°C)
Cold exposure (h)
b
WT (n=12)
Sirt7 KO (n=9)
VO2 (mL/kg/min)
Zeitgeber time
c
WT (n=12)
Sirt7 KO (n=9)
Energy Expenditure (kcal/kg/day)
Dark
Light
Total
d
Body temp. (°C)
WT (n=12)
Sirt7 KO (n=9)
e
WT (n=8)
Sirt7 KO (n=8)
VO2 (mL/kg/min)
Zeitgeber time
f
WT (n=8)
Sirt7 KO (n=8)
Energy Expenditure (kcal/kg/day)
Dark
Light
Total
g
Body temp. (°C)
WT (n=8)
Sirt7 KO (n=8)

Supplementary Fig. 1 ***Sirt7*** **KO mice display increased energy expenditure and body temperature under normal conditions.**

a Body temperature in 11-week-old male WT and *Sirt7* KO mice upon cold exposure at 4°C. $p = 0.0328$ (0 h), $p = 0.0485$ (3 h), $p = 0.0018$ (5 h). **b–d** Data of indirect calorimetry experiments and body temperature from 16-week-old female WT and *Sirt7* KO mice. VO_2 rates (**b**), energy expenditure (**c**), and body temperature at ZT10 (**d**). $p = 0.0483$ in (**b**); $p = 0.0466$ (Dark) in (**c**); $p = 0.0135$ in (**d**). **e–g** Data of indirect calorimetry experiments and body temperature from 2-month-old male WT and another independent line of *Sirt7* KO mice[35]. VO_2 rates (**e**), energy expenditure (**f**), and body temperature at ZT10 (**g**). $p = 0.0323$ in (**e**); $p = 0.0503$ (Dark), $p = 0.0288$ (Light), $p = 0.0350$ (Total) in (**f**); $p = 0.0242$ in (**g**). Data are presented as means ± SEM. All numbers (n) are biologically independent samples. Two-way ANOVA with Bonferroni's multiple comparisons test (**b, e**); two-tailed Student's *t*-test (**a**, **c**, **d**, **f**, **g**). *$p < 0.05$, **$p < 0.01$. Source data are provided in Source data file.

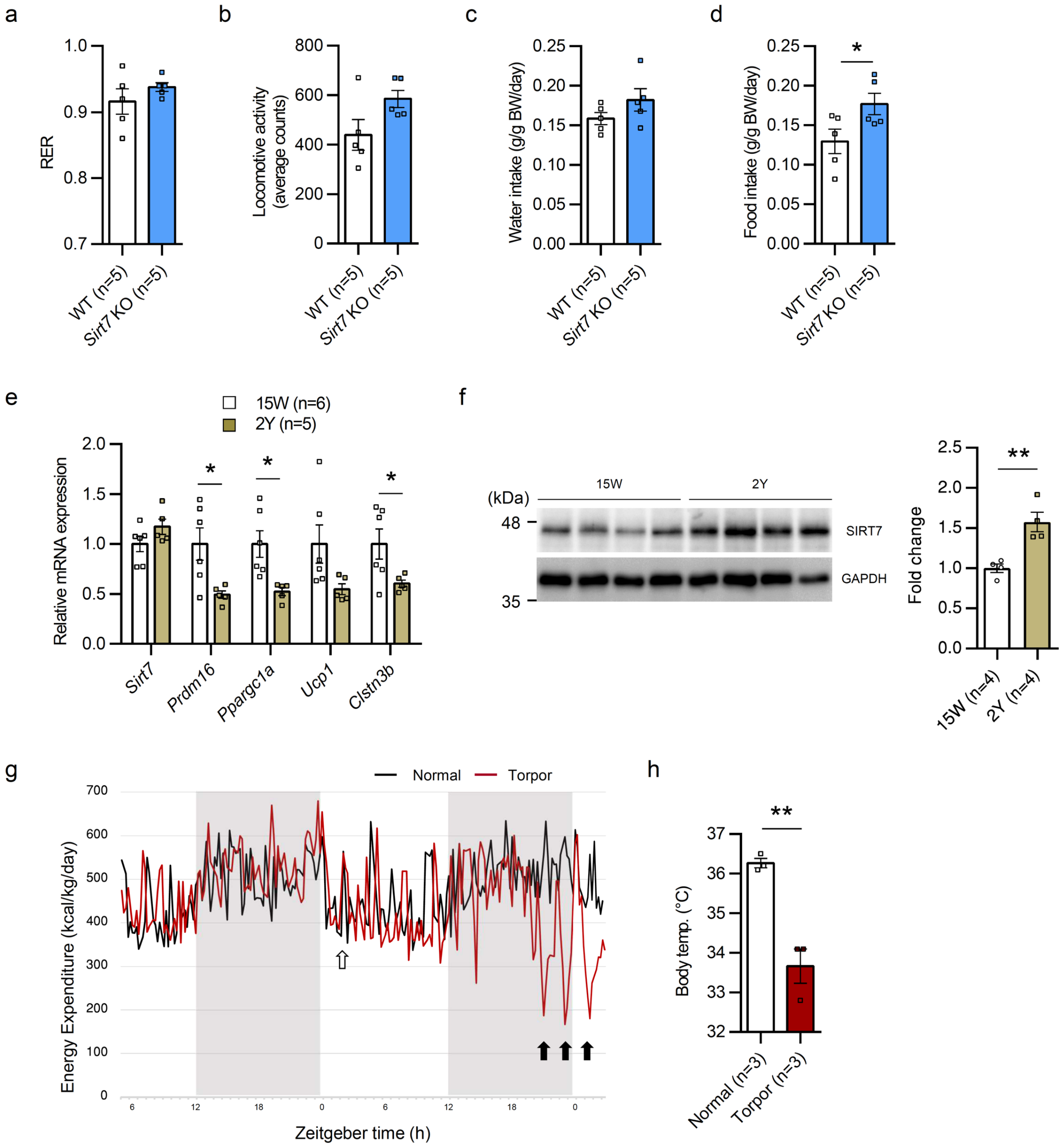

a
RER
WT (n=5)
Sirt7 KO (n=5)
b
Locomotive activity (average counts)
c
Water intake (g/g BW/day)
d
Food intake (g/g BW/day)
e
15W (n=6)
2Y (n=5)
Relative mRNA expression
Sirt7
Prdm16
Ppargc1a
Ucp1
Clstn3b
f
(kDa)
15W
2Y
SIRT7
GAPDH
Fold change
15W (n=4)
2Y (n=4)
g
Normal
Torpor
Energy Expenditure (kcal/kg/day)
Zeitgeber time (h)
h
Body temp. (°C)
Normal (n=3)
Torpor (n=3)

Supplementary Fig. 2 ***Sirt7*** **KO mice exhibit excessive energy expenditure and thermogenesis in the hypometabolic state.**

a–d Data of indirect calorimetry experiments from 2-year-old male WT and *Sirt7* KO mice. RER (**a**), locomotor activity (**b**), water intake (**c**), and food intake (**d**). $p = 0.0488$ in (**d**). **e** Real-time qPCR analysis of BAT-related genes in iBAT of 15-week-old and 2-year-old male WT mice. $p = 0.0250$ (*Prdm16*), $p = 0.0153$ (*Ppargc1a*), $p = 0.0664$ (*Ucp1*), $p = 0.0464$ (*Clstn3b*). **f** Western blot analysis of SIRT7 in iBAT of 15-week-old and 2-year-old male WT mice. $p = 0.0050$. **g**, **h** Data of indirect calorimetry experiments and body temperature during artificially-induced daily torpor in 18-week-old male WT mice. Representative pattern of energy expenditure (**g**). The mouse was placed in the chamber at 20°C for 3 days; food was removed on the second day (open arrow) in the Torpor groups. A typical daily torpor pattern started after 20 h of fasting (filled arrow). Body temperature after 24 h fasting (**h**). $p = 0.0044$. Data are presented as means ± SEM. All numbers (n) are biologically independent samples. $*p < 0.05$, $**p < 0.01$ by two-tailed Student's t-test. Source data are provided in Source data file.

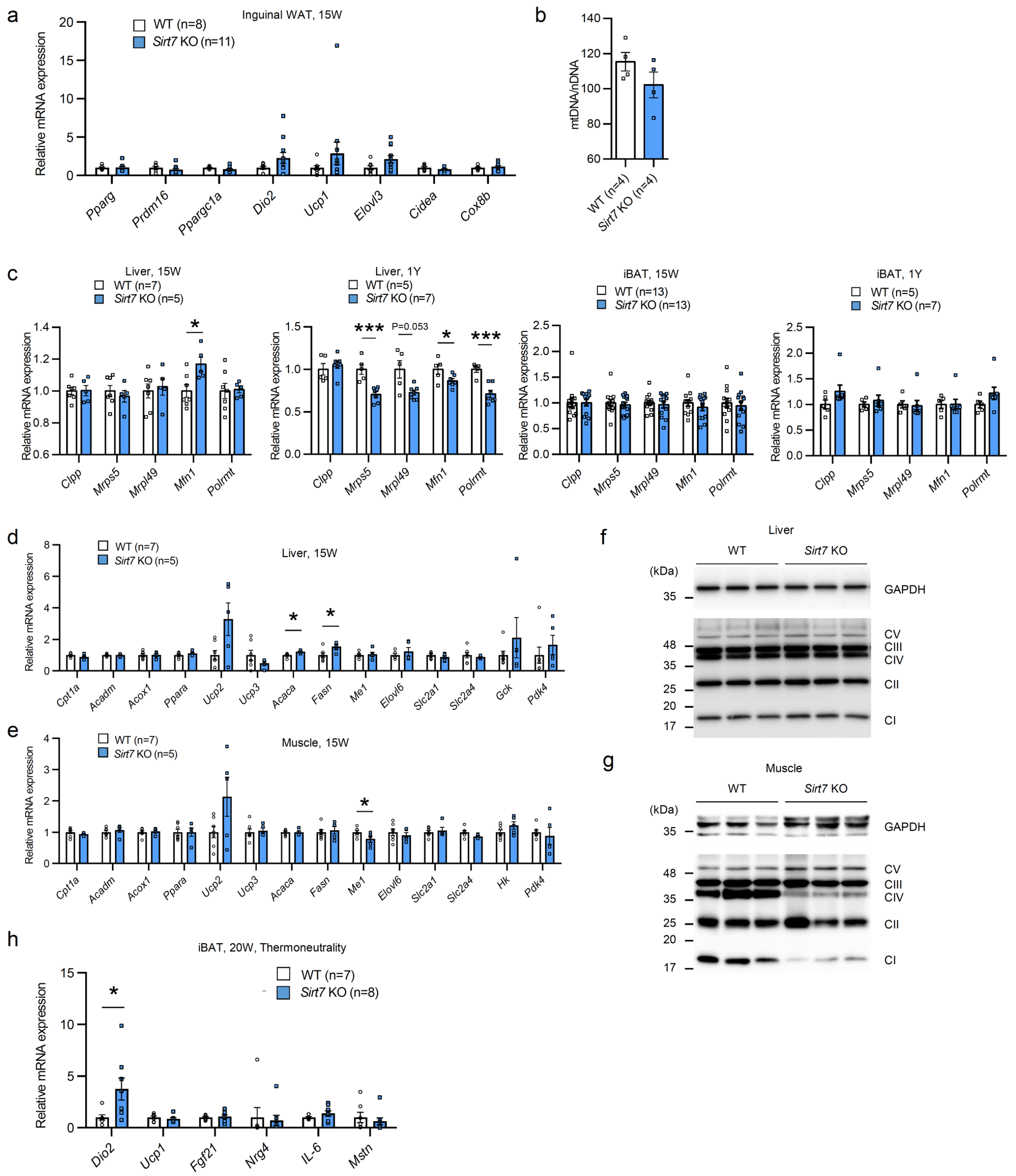
a
Inguinal WAT, 15W
WT (n=8)
Sirt7 KO (n=11)
Relative mRNA expression
Pparg
Prdm16
Ppargc1a
Dio2
Ucp1
Elovl3
Cidea
Cox8b
b
mtDNA/nDNA
WT (n=4)
Sirt7 KO (n=4)
c
Liver, 15W
WT (n=7)
Sirt7 KO (n=5)
Liver, 1Y
WT (n=5)
Sirt7 KO (n=7)
P=0.053
iBAT, 15W
WT (n=13)
Sirt7 KO (n=13)
iBAT, 1Y
WT (n=5)
Sirt7 KO (n=7)
Clpp
Mrps5
Mrpl49
Mfn1
Polrmt
d
Liver, 15W
WT (n=7)
Sirt7 KO (n=5)
Cpt1a
Acadm
Acox1
Ppara
Ucp2
Ucp3
Acaca
Fasn
Me1
Elovl6
Slc2a1
Slc2a4
Gck
Pdk4
e
Muscle, 15W
WT (n=7)
Sirt7 KO (n=5)
Hk
f
Liver
WT
Sirt7 KO
(kDa)
GAPDH
CV
CIII
CIV
CII
CI
g
Muscle
h
iBAT, 20W, Thermoneutrality
WT (n=7)
Sirt7 KO (n=8)
Dio2
Ucp1
Fgf21
Nrg4
IL-6
Mstn

Supplementary Fig. 3 SIRT7 suppresses energy expenditure and thermogenesis via multiple pathways.

a Real-time qPCR analysis of BAT-related genes in inguinal WAT of 15-week-old male WT and *Sirt7* KO mice. **b** Evaluation of the mtDNA copy number in iBAT of 15-week-old male WT and *Sirt7* KO mice by determining the ratio of mtDNA to nuclear DNA (nDNA). **c** Real-time qPCR analysis of nuclear-encoded mitochondrial transcripts in liver and iBAT of 15-week-old and 1-year-old male WT and *Sirt7* KO mice. The expression of the mitochondrial unfolded protein response gene *Clpp* is increased in hematopoietic stem cells isolated from *Sirt7* KO mice[45], and the expression of genes related to mitochondrial biogenesis, namely, *Mrps5*, *Mrpl49*, *Mfn1*, and *Polrmt*, is reduced in the liver of aged *Sirt7* KO mice[35]. $p = 0.0174$ (*Mfn1*) in liver, 15 weeks. $p = 0.0009$ (*Mrps5*), $p = 0.0525$ (*Mrpl49*), $p = 0.0424$ (*Mfn1*), $p = 0.0005$ (*Polrmt*) in liver, 1 year. **d, e** Real-time qPCR analysis of energy metabolism-related genes in liver (**d**) and skeletal muscle (**e**) of 15-week-old male WT and *Sirt7* KO mice. $p = 0.0101$ (*Acaca*), $p = 0.0269$ (*Fasn*) in (**d**); $p = 0.0499$ (*Me1*) in (**e**). **f, g** Western blot analysis of OXPHOS complexes I–IV in liver (**f**) and skeletal muscle (**g**) of 15-week-old male WT and *Sirt7* KO mice. **h** Real-time qPCR analysis of batokine genes in iBAT of 20-week-old male WT and *Sirt7* KO mice at thermoneutrality. $p = 0.0375$ (*Dio2*). Data are presented as means ± SEM. All numbers (n) are biologically independent samples. $*p < 0.05$, $**p < 0.01$, $***p < 0.001$ by two-tailed Student's *t*-test. Source data are provided in Source data file.

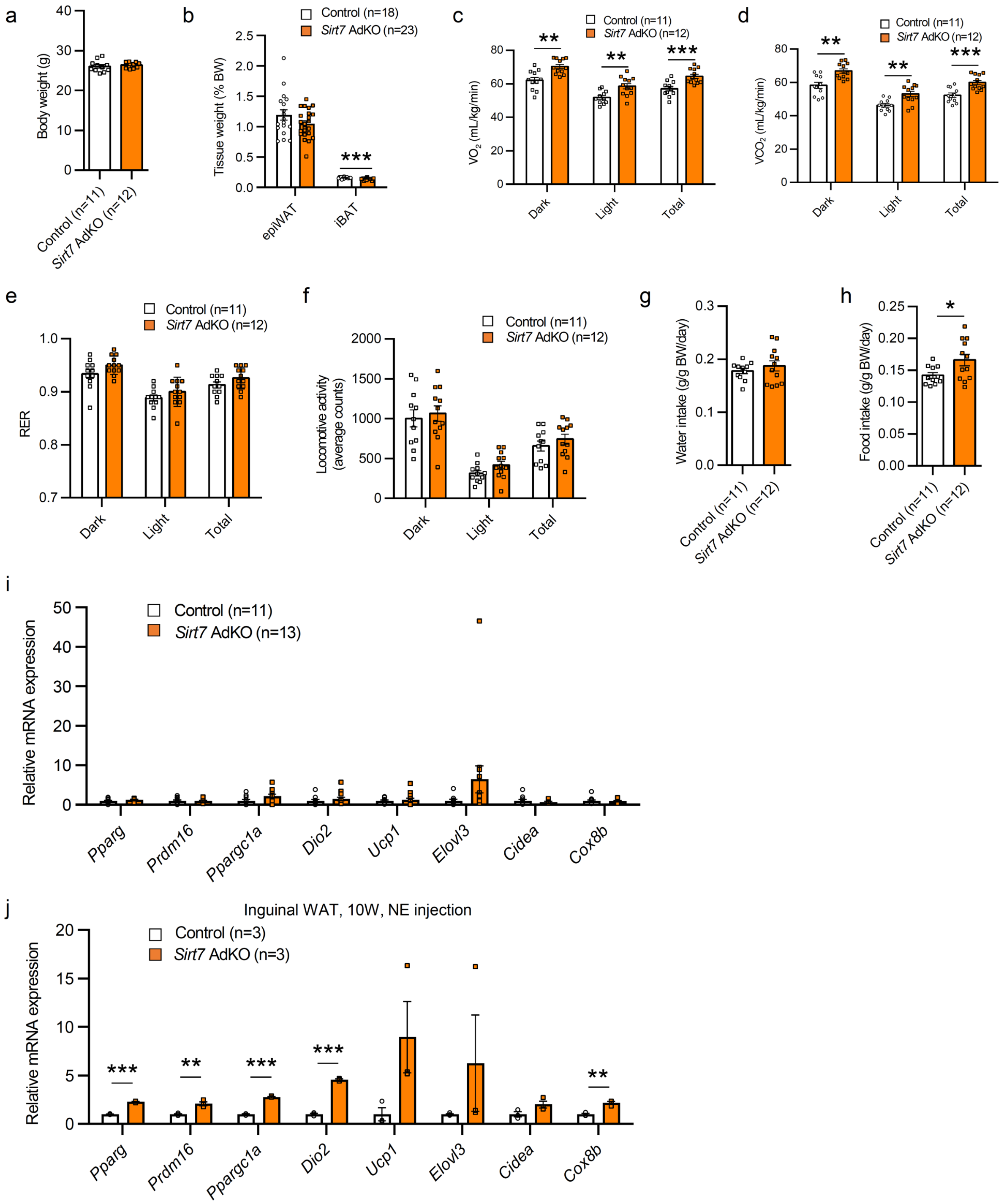
a
Body weight (g)
Control (n=11)
Sirt7 AdKO (n=12)
b
Control (n=18)
Sirt7 AdKO (n=23)
Tissue weight (% BW)
epiWAT
iBAT
***
c
Control (n=11)
Sirt7 AdKO (n=12)
VO2 (mL/kg/min)
Dark
Light
Total
d
Control (n=11)
Sirt7 AdKO (n=12)
VCO2 (mL/kg/min)
Dark
Light
Total
e
Control (n=11)
Sirt7 AdKO (n=12)
RER
Dark
Light
Total
f
Control (n=11)
Sirt7 AdKO (n=12)
Locomotive activity (average counts)
Dark
Light
Total
g
Water intake (g/g BW/day)
Control (n=11)
Sirt7 AdKO (n=12)
h
Food intake (g/g BW/day)
Control (n=11)
Sirt7 AdKO (n=12)
i
Control (n=11)
Sirt7 AdKO (n=13)
Relative mRNA expression
Pparg
Prdm16
Ppargc1a
Dio2
Ucp1
Elovl3
Cidea
Cox8b
j
Inguinal WAT, 10W, NE injection
Control (n=3)
Sirt7 AdKO (n=3)
Relative mRNA expression
Pparg
Prdm16
Ppargc1a
Dio2
Ucp1
Elovl3
Cidea
Cox8b

Supplementary Fig. 4 SIRT7 deficiency in adipose tissue elevates the whole-body energy expenditure and body temperature.

a–h Data of indirect calorimetry experiments from 12-week-old male *Adipoq-Cre* control and *Sirt7* AdKO mice. Body weight (**a**), percent tissue weight calculated relative to body weight (**b**), average VO_2 (**c**), average VCO_2 (**d**), RER (**e**), locomotor activity (**f**), water intake (**g**), and food intake (**h**). $p = 0.0007$ (iBAT) in (**b**); $p = 0.0012$ (Dark), $p = 0.0033$ (Light), $p = 0.0007$ (Total) in (**c**); $p = 0.0010$ (Dark), $p = 0.0025$ (Light), $p = 0.0004$ (Total) in (**d**); $p = 0.0279$ in (**h**). **i** Real-time qPCR analysis of BAT-related genes in inguinal WAT of 12-week-old male *Adipoq-Cre* control and *Sirt7* AdKO mice. **j** Real-time qPCR analysis of BAT-related genes in inguinal WAT of 10-week-old male *Adipoq-Cre* control and *Sirt7* AdKO mice administered 1 mg/kg/day norepinephrine (NE) for 5 days. p = 2.7E–05 (*Pparg*), $p = 0.0077$ (*Prdm16*), p = 2.8E–05 (*Ppargc1a*), p = 9.7E–06 (*Dio2*), $p = 0.0039$ (*Cox8b*). Data are presented as means ± SEM. All numbers (n) are biologically independent samples. *$p < 0.05$, **$p < 0.01$, ***$p < 0.001$ by two-tailed Student's *t*-test. Source data are provided in Source data file.

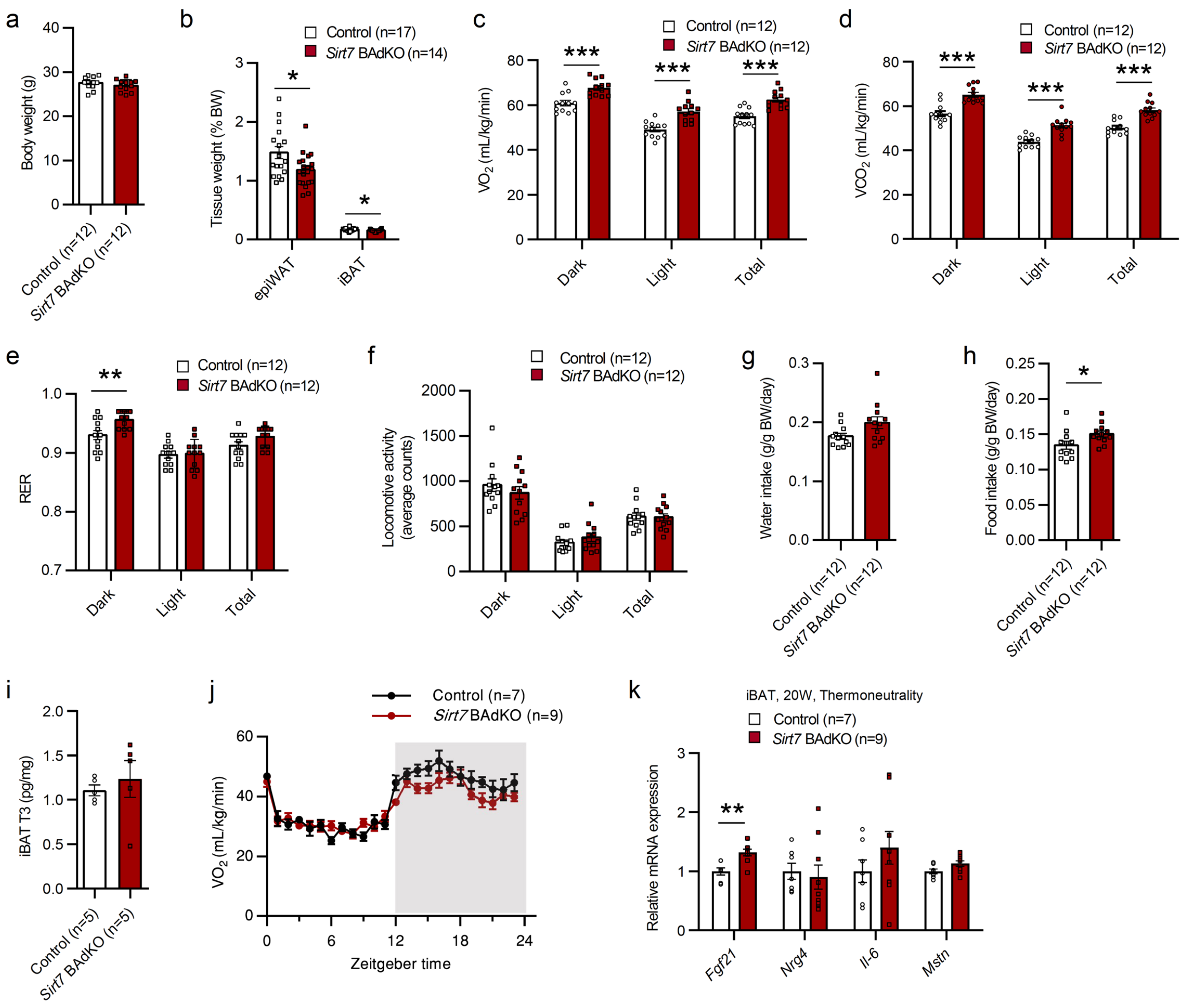
a
Body weight (g)
Control (n=12)
Sirt7 BAdKO (n=12)
b
Control (n=17)
Sirt7 BAdKO (n=14)
Tissue weight (% BW)
epiWAT
iBAT
c
Control (n=12)
Sirt7 BAdKO (n=12)
VO2 (mL/kg/min)
Dark
Light
Total
d
Control (n=12)
Sirt7 BAdKO (n=12)
VCO2 (mL/kg/min)
e
Control (n=12)
Sirt7 BAdKO (n=12)
RER
f
Control (n=12)
Sirt7 BAdKO (n=12)
Locomotive activity (average counts)
g
Water intake (g/g BW/day)
h
Food intake (g/g BW/day)
i
iBAT T3 (pg/mg)
Control (n=5)
Sirt7 BAdKO (n=5)
j
Control (n=7)
Sirt7 BAdKO (n=9)
VO2 (mL/kg/min)
Zeitgeber time
k
iBAT, 20W, Thermoneutrality
Control (n=7)
Sirt7 BAdKO (n=9)
Relative mRNA expression
Fgf21
Nrg4
Il-6
Mstn

Supplementary Fig. 5 Brown adipocytic SIRT7 suppresses energy expenditure and thermogenesis *in vivo*.

a–h Data of indirect calorimetry experiments from 12-week-old male *Ucp1-Cre* control and *Sirt7* BAdKO mice. Body weight (**a**), percent tissue weight calculated relative to body weight (**b**), average VO_2 (**c**), average VCO_2 (**d**), RER (**e**), locomotor activity (**f**), water intake (**g**), and food intake (**h**). $p = 0.0428$ (epiWAT), $p = 0.0474$ (iBAT) in (**b**); $p = 0.0004$ (Dark), $p = 0.0001$ (Light), $p = 0.0001$ (Total) in (**c**); $p = 2.7E–05$ (Dark), $p = 8.3E–06$ (Light), $p = 5.0E–06$ (Total) in (**d**); $p = 0.0087$ (Dark) in (**e**); $p = 0.0335$ in (**h**). **i** T3 level in iBAT of 12-week-old male *Ucp1-Cre* control and *Sirt7* BAdKO mice. **j**, **k** VO_2 rates of indirect calorimetry experiments (**j**) and real-time qPCR analysis of batokine genes in iBAT (**k**) from 20-week-old male *Ucp1-Cre* control and *Sirt7* BAdKO mice at thermoneutrality. $p = 0.0016$ (*Fgf21*). Data are presented as means ± SEM. All numbers (n) are biologically independent samples. Two-way ANOVA with Bonferroni's multiple comparisons test (**j**); two-tailed Student's *t*-test (**a–i**, **k**). *$p < 0.05$, **$p < 0.01$, ***$p < 0.001$. Source data are provided in Source data file.

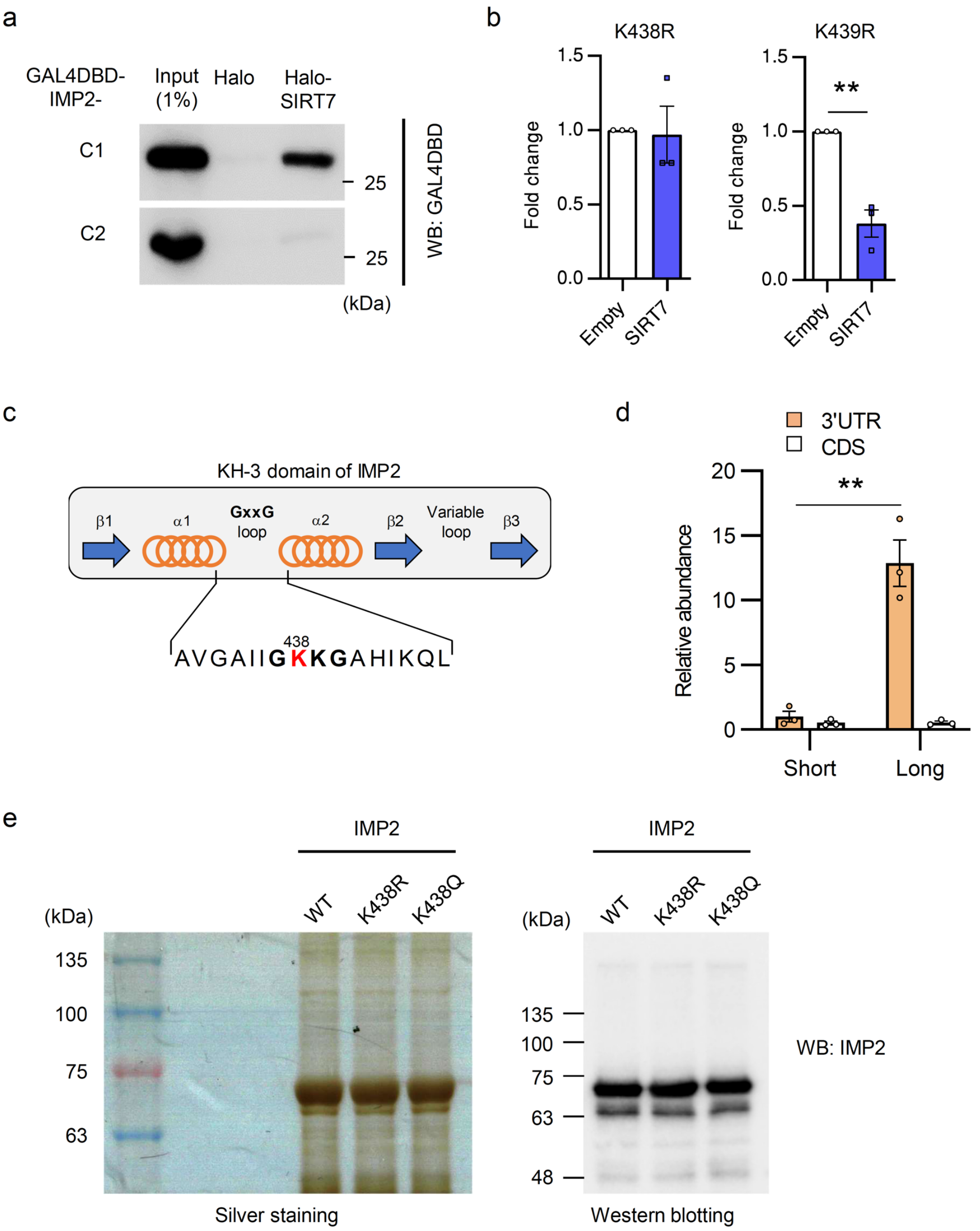

f

Proteins containing KH domain

AKAP1; ANKHD1; ANKRD17; ASCC1; BICC1; DDX43; DDX53; DPPA5; FMR1; FUBP1; FUBP3; FXR1; FXR2; GLD1; HDLBP; HNRPK; IGF2BP1; IGF2BP2; IGF2BP3; KHDRBS1; KHDRBS2; KHDRBS3; KHSRP; KRR1; MEX3A; MEX3B; MEX3C; MEX3D; NOVA1; NOVA2; PCBP1; PCBP2; PCBP3; PCBP4; PNO1; PNPT1; QKI; SF1; TDRKH

Supplementary Fig. 6 Deacetylation of IMP2 by SIRT7 attenuates the translation of *Ucp1* mRNA.

a Mapping of the region of the interaction between IMP2 and SIRT7. Halo-SIRT7 pull-down assay with lysates of HEK293T cells expressing the indicated IMP2 deletion mutants fused with GAL4DBD. See also Fig. 8c. **b** Quantification of the acetylated IMP2 relative to IMP2 (n = 3) in the experiment of Fig. 8d. $p = 0.0025$. **c** Topology of the KH-3 domain and amino acid sequence around K438 of mouse IMP2. **d** Binding of IMP2 to *in vitro*-transcribed short and long *Ucp1* 3´-UTR (n = 3 independent samples per group). The *Ucp1* coding sequence (CDS) was used as a negative control. $p = 0.0030$. **e** Generation of recombinant protein in *E. coli* for $IMP2^{WT}$, $IMP2^{K438R}$, and $IMP2^{K438Q}$. To confirm that equal amounts of these recombinant proteins were used in the insect cell-free translation system, silver staining and WB were performed. This confirmation was performed once. **f** Candidate SIRT7-interacting proteins in KH domain family. The 10 proteins marked in red have been reported to be candidate SIRT7-interacting proteins[49]. See Discussion for details. WB, western blotting. Data are presented as means ± SEM. All numbers (n) are biologically independent samples. $*p < 0.05$, $**p < 0.01$ by two-tailed Student's *t*-test. Source data are provided in Source data file.

qPCR primer pairs for mouse mRNA			
**Symbol**	**Name**	**Sequence**	
		**Forward**	**Reverse**
Rpl19	ribosomal protein L19	AAGCCTGTGACTGTCCATTC	CTTCTTGGATTCCCGGTATC
Pparg	peroxisome proliferator-activated receptor gamma	GGAAGACCACTCGCATTCCTT	TCGCACTTTGGTATTCTTGGAG
Prdm16	PR domain containing 16	GGCGAGGAAGCTAGCCAAA	GGTCTCCTCCTCGGCACTCT
Ppargc1a	peroxisome proliferative activated receptor, gamma, coactivator 1 alpha	GAAATCCGAGCGGAGCTGAA	GAATAGGGCTGCGTGCCATC
Cebpb	CCAAT/enhancer binding protein (C/EBP), beta	CAACCTGGAGACGCAGCACAAG	CTAGCAGTGGCCCGCCGAGG
Ebf2	early B cell factor 2	GCTGCGGGAACCGGAACGAGA	ACACGACCTGGAACCGCCTCA
Dio2	deiodinase, iodothyronine, type II	AGTCAAGAAGGTGGCATTCG	ACAGCTTCCTCCTAGATGCCT
Ucp1	uncoupling protein 1	GGCAACAAGAGCTGACAGTAAAT	GGCCCTTGTAAACAACAAAATAC
Elovl3	elongation of very long chain fatty acids-like 3	TTGGGGATAGGGGGTGTGTG	TCTCCCCTCCCCTCCAAGTC
Cidea	cell death-inducing DFFA-like effector a	CTTGGGGGTGGTACCCAGTG	ATCCACGCAGTTCCCACACA
Cox3	mitochondrially encoded cytochrome c oxidase III	GCAGGATTCTTCTGAGCGTTCT	GTCAGCAGCCTCCTAGATCATGT
Cox8b	cytochrome c oxidase subunit 8B	GAACCATGAAGCCAACGACT	GCGAAGTTCACAGTGGTTCC
Clstn3b	calsyntenin 3	CTCCGCAGGAACAGCAGCCC	AGGATAACCATAAGCACCAG
S100b	S100 protein, beta polypeptide, neural	TGGTTGCCCTCATTGATGTCT	CCCATCCCCATCTTCGTCC
Bmp8b	bone morphogenetic protein 8b	CAACCACGCCACTATGCAG	CACTCAGCTCAGTAGGCACA
Fgf21	fibroblast growth factor 21	GCCATTCACTTTGCCTGAGC	ATCCATTCCATCAGGGCTGC
Nrg4	neuregulin 4	ATGCCAACAGATCACGAGC	TCTTCAGTGTTCTCTGTGGCTG
Il-6	interleukin 6	CCACGGCCTTCCCTACTTCA	TTCCACGATTTCCCAGAGAACA
Mstn	myostatin	AGTGGATCTAAATGAGGGCAGT	GTTTCCAGGCGCAGCTTAC
Sirt7	sirtuin 7	TGCCAGGCACTTGGTTGTCT	TAGGCTCCGCTTCGCTTAGG
Clpp	caseinolytic mitochondrial matrix peptidase proteolytic subunit	TGTTGCGGGAACGCATCGTGT	TAGATGGCCAGGCCCGCAGTT
Mrps5	mitochondrial ribosomal protein S5	TCCCTGCATTGTGTAGCTTG	GAGCTGTCTTCACTGCCAAA
Mrpl49	mitochondrial ribosomal protein L49	GAGCGACGAACAAAGTAGGG	TACCAAAATCCCAGAGCCAC
Mfn1	mitofusin 1	AGGGGACCGATGGAGATAAAG	AAGAGGGCACATTTTGCTTTG
Polrmt	polymerase (RNA) mitochondrial (DNA directed)	GCAACATGTCCTGAGGGAGT	GCACCTTCTTCACCCTCATC
Cpt1a	carnitine palmitoyltransferase 1a, liver	CCATGATGGACCCCACAACA	TGGACAACCTCCATGGCTCA
Acadm	acyl-Coenzyme A dehydrogenase, medium chain	GCAGCTGGCGCTGTCGGGCT	CGGCGTCAGTGGCTAGCTGATTG
Acox1	acyl-Coenzyme A oxidase 1, palmitoyl	GGGAGTGCTACGGGTTACATG	CCGATATCCCCAACAGTGATG
Ppara	peroxisome proliferator activated receptor alpha	TTGAAGGAGCTTTGGGAAGA	AGGAAGCCGTTCTGTGACAT
Ucp2	uncoupling protein 2 (mitochondrial, proton carrier)	GAGGGTCCACGCAGCCTCTA	GCATGCTCTGAGCCCTTGGT
Ucp3	uncoupling protein 3 (mitochondrial, proton carrier)	GTGGCCCTGCACTACCCAAC	CGGTGTCCAGGGGAAAAGTG
Acaca	acetyl-Coenzyme A carboxylase alpha	CCAGCTGATCCTGCGAACCT	GAACATTCCCGCAAGCCATC
Fasn	fatty acid synthase	TCTGGGCCAACCTCATTGGT	GAAGCTGGGGGTCCATTGTG
Me1	malic enzyme 1, NADP(+)-dependent, cytosolic	CTGGCCAGGGCAACAATTCC	TAGAGCCGGCCTTCTTGCAG
Elovl6	ELOVL family member 6, elongation of long chain fatty acids	GGAGCAGAGGCGCAGAGAAC	GAGCGGCTTCCGAAGTTCAA
Slc2a1	solute carrier family 2 (facilitated glucose transporter), member 1	GCTTATGGGCTTCTCCAAACT	GGTGACACCTCTCCCACATAC
Slc2a4	solute carrier family 2 (facilitated glucose transporter), member 4	ACCCCTCATTCCCCCTGTGT	ACCCTCCTGCAGACCCCTTC
Gck	glucokinase	CCCACAAATGCTCCCAGTCC	GTGGGAGGCCAGAAGCTGAA
Pdk4	pyruvate dehydrogenase kinase, isoenzyme 4	CGCGCTCCTGACCCGCAGCC	GCCAGGCGGACGGGCAGCTC
Hk1	hexokinase 1	TGCCTCTGGGCTTCACCTTC	CCACACAGTCGGTGGCTTTG
Ucp1 3´-UTR	uncoupling protein 1, 3´-UTR	GCAACTTGGAGGAAGAGATA	GATTTCTTTGGTTGGTTTTATTC
Ucp1 CDS	uncoupling protein 1, coding sequence	GGGCCCTTGTAAACAACAAA	GTCGGTCCTTCCTTGGTGTA

qPCR primer pairs for mouse genome

Symbol	Name	Sequence	
		Forward	Reverse
mt-Nd1	NADH dehydrogenase 1, mitochondrial	CCTATCACCCTTGCCATCAT	GAGGCTGTTGCTTGTGTGAC
Pecam1	platelet/endothelial cell adhesion molecule 1	ATGGAAAGCCTGCCATCATG	TCCTTGTTGTTCAGCATCAC

Cloning primer pairs for cDNA

Symbol	Forward	Reverse
Imp2	agaattcATGATGAACAAGCTGTACATTGGG	actcgagTCACTTGCTGCGCTGTGGGGCGACTCCC

Guide oligonucleotides for sgRNAs

Symbol	Forward	Reverse
Imp2	caccgGGGAACAAGGCCACGGCCCC	aaacgGGGCCGTGGCCTTGTTCCCC

Supplementary Table 1 Information of primers.

Name	Host & Clonality / Source & Identifier	Dilution
anti-UCP1	Rabbit Polyclonal / Abcam, ab10983	1:1000 dilution in TBST 5% BSA
anti-GAPDH	Rabbit Monoclonal (14C10) / Cell Signaling Technology, #2118	1:1000 dilution in TBST 5% BSA
Total OXPHOS Rodent WB Antibody Cocktail	Mouse Monoclonal (20E9DH10C12, 21A11AE7, 13G12AF12BB11, 1D6E1A8, 15H4C4) / Abcam, ab110413	1:250 dilution in TBST 1% skim milk
anti-Tyrosine Hydroxylase	Sheep Polyclonal / Sigma-Aldrich, AB1542	1:1000 dilution in TBST 5% BSA
anti-RNA Pol II	Mouse Monoclonal (4H8) / Active Motif, #39097	1:2000 dilution in TBST 3% skim milk
anti-SIRT7	Rabbit Monoclonal (D3K5A) / Cell Signaling Technology, #5360	1:3000 dilution in TBST 5% BSA
anti-IGFBP2(IMP2)	Rabbit Polyclonal / MBL, #RN008P	1:1000 dilution in TBST 5% BSA
anti-DYKDDDDK (FLAG) tag	Mouse Monoclonal (1E6) / Fujifilm Wako Pure Chemical Corporation, #018-22381	1:2000 dilution in TBST 3% skim milk
anti-HA tag	Rat Monoclonal (3F10) / Roche Applied Science, #11867423001	1:1000 dilution in TBST 5% BSA
anti-acetylated-lysine	Rabbit Polyclonal / Cell Signaling Technology, #9441	1:1000 dilution in TBST 5% BSA
HRP-conjugated Affinipure Goat anti-Rabbit IgG(H+L)	Goat Polyclonal / Proteintech, #SA00001-2	1:10000 dilution in TBST 5% skim milk
HRP-conjugated Affinipure Goat anti-Mouse IgG(H+L)	Goat Polyclonal / Proteintech, #SA00001-1	1:10000 dilution in TBST 5% skim milk
HRP-conjugated Affinipure Goat anti-Rat IgG(H+L)	Goat Polyclonal / Proteintech, #SA00001-15	1:10000 dilution in TBST 5% skim milk
HRP-conjugated Affinipure Rabbit anti-Sheep IgG(H+L)	Rabbit Polyclonal / Proteintech, #SA00001-16	1:10000 dilution in TBST 5% skim milk

Supplementary Table 2 Information of antibodies.

All antibodies used in this study are commercially available and have been validated by companies. The details of manufactures' validation can be found on the web site of each manufacture. See also the Reporting Summary.
