# Supplementary material for: SIRT7 suppresses energy expenditure and thermogenesis by regulating brown adipose tissue functions in mice
Source: Nat Commun. 2022 Dec 12;13:7439. doi: 10.1038/s41467-022-35219-z (PMC9744749; doi:10.1038/s41467-022-35219-z)
Supplement: Supplementary file 3 — Description of Additional Supplementary Files [file 41467_2022_35219_MOESM3_ESM.pdf]

Description of Additional Supplementary Files

File Name: Supplementary Data 1
Description: The candidate molecules interacting with SIRT7. After Halo-SIRT7 pull-down assay, eluted proteins were resolved by SDS-PAGE, followed by silver staining (Fig. 7a). The gel lanes were cut into 26 pieces and the proteins within each gel piece were analyzed by LC-MS/MS analysis.
