# Supplementary material for: SIRT7 suppresses energy expenditure and thermogenesis by regulating brown adipose tissue functions in mice
Source: Nat Commun. 2022 Dec 12;13:7439. doi: 10.1038/s41467-022-35219-z (PMC9744749; doi:10.1038/s41467-022-35219-z)
Supplement: Supplementary file 5 — Reporting Summary [file 41467_2022_35219_MOESM5_ESM.pdf]

Corresponding author(s): Tatsuya Yoshizawa and Kazuya Yamagata

Last updated by author(s): Nov 4, 2022

Reporting Summary

Nature Portfolio wishes to improve the reproducibility of the work that we publish. This form provides structure for consistency and transparency in reporting. For further information on Nature Portfolio policies, see our Editorial Policies and the Editorial Policy Checklist.

Statistics

For all statistical analyses, confirm that the following items are present in the figure legend, table legend, main text, or Methods section.

n/a	Confirmed	
☐	☒	The exact sample size (*n*) for each experimental group/condition, given as a discrete number and unit of measurement
☐	☒	A statement on whether measurements were taken from distinct samples or whether the same sample was measured repeatedly
☐	☒	The statistical test(s) used AND whether they are one- or two-sided<br>*Only common tests should be described solely by name; describe more complex techniques in the Methods section.*
☒	☐	A description of all covariates tested
☐	☒	A description of any assumptions or corrections, such as tests of normality and adjustment for multiple comparisons
☐	☒	A full description of the statistical parameters including central tendency (e.g. means) or other basic estimates (e.g. regression coefficient) AND variation (e.g. standard deviation) or associated estimates of uncertainty (e.g. confidence intervals)
☐	☒	For null hypothesis testing, the test statistic (e.g. *F*, *t*, *r*) with confidence intervals, effect sizes, degrees of freedom and *P* value noted<br>*Give P values as exact values whenever suitable.*
☒	☐	For Bayesian analysis, information on the choice of priors and Markov chain Monte Carlo settings
☒	☐	For hierarchical and complex designs, identification of the appropriate level for tests and full reporting of outcomes
☒	☐	Estimates of effect sizes (e.g. Cohen's *d*, Pearson's *r*), indicating how they were calculated

Our web collection on statistics for biologists contains articles on many of the points above.

Software and code

Policy information about availability of computer code

Data collection	Metabolic measuring system (Model MK-5000, Muromachi Kikai, Japan) was used to collect the data of indirect calorimetry experiments.<br>Real-time qPCR was performed using an Applied Biosystems 7300 or ViiA7 thermal cycler (Thermo Fisher Scientific).<br>Microplate reader (iMark 168-1130, BIO-RAD) was used for ELISA.<br>The OCR of cells was measured using the XF24 Extracellular Flux Analyzer (Seahorse Bioscience).<br>The peptide solution was analyzed by a LC-MS/MS system (LTQ Velos Pro, Thermo Fisher Scientific).
Data analysis	Lipid areas were quantified by using ImageJ 1.45s.<br>The protein bands were quantified by Bio-Rad Image Lab 6.0 software.<br>All MS/MS spectral data were analyzed by Proteome Discoverer 1.3.<br>All data were analyzed using appropriate statistical methods with GraphPad Prism 9.

For manuscripts utilizing custom algorithms or software that are central to the research but not yet described in published literature, software must be made available to editors and reviewers. We strongly encourage code deposition in a community repository (e.g. GitHub). See the Nature Portfolio guidelines for submitting code & software for further information.

Data

Policy information about availability of data

All manuscripts must include a data availability statement. This statement should provide the following information, where applicable:

- Accession codes, unique identifiers, or web links for publicly available datasets
- A description of any restrictions on data availability
- For clinical datasets or third party data, please ensure that the statement adheres to our policy

All MS/MS spectral data were searched against entries for mice in the Swiss-Prot database (v2013-01-04) using the SEQUEST database search program using Proteome Discoverer 1.3. The mass spectrometry proteomics data have been deposited at jPOSTrepo (https://repository.jpostdb.org/) and are publicly available under accession ID (ID: JPST001343/PXD028870). All the other data supporting this study are available within this Article, Supplementary Information, and Source data. Source data are provided with this paper.

Field-specific reporting

Please select the one below that is the best fit for your research. If you are not sure, read the appropriate sections before making your selection.

☒ Life sciences ☐ Behavioural & social sciences ☐ Ecological, evolutionary & environmental sciences

For a reference copy of the document with all sections, see nature.com/documents/nr-reporting-summary-flat.pdf

Life sciences study design

All studies must disclose on these points even when the disclosure is negative.

Sample size	No statistical methods were used to determine sample size, but the sample sizes were similar to those of previous reports (ref. 33,35).
Data exclusions	No data were excluded.
Replication	The number of biological replicates is reported in the figure legend.
Randomization	Mice were randomly assigned into separate group. There was no randomization for in vitro experiments due to the need to identify specific genotypes or treat cells with chemicals or DNA according to experimental designs, but the experiments were conducted and checked by the other researcher.
Blinding	Investigators were blinded to group allocation during data collection of body temperature. No mice experiments concerning energy metabolism required an analysis for which bias may alter the results, because the data acquisition was performed by machines automatically without human intervention. There was no blinding for in vitro cellular experiment due to the need to identify specific genotypes or treat cells with chemicals according to experimental designs. Investigators were blinded to sample identity during data collection of ELISA and histology. Blinding was not possible for biochemical experiments which require sample loading in appropriate orders for western blot, but analysis results were confirmed by the other researcher.

Reporting for specific materials, systems and methods

We require information from authors about some types of materials, experimental systems and methods used in many studies. Here, indicate whether each material, system or method listed is relevant to your study. If you are not sure if a list item applies to your research, read the appropriate section before selecting a response.

Materials & experimental systems

n/a	Involved in the study	
☐	☒	Antibodies
☐	☒	Eukaryotic cell lines
☒	☐	Palaeontology and archaeology
☐	☒	Animals and other organisms
☒	☐	Human research participants
☒	☐	Clinical data
☒	☐	Dual use research of concern

Methods

n/a	Involved in the study	
☒	☐	ChIP-seq
☒	☐	Flow cytometry
☒	☐	MRI-based neuroimaging

Antibodies

Antibodies used	All antibodies used in this study are commercially available. Please also see Methods and Supplemaentary Table 2 for further description of antibodies, including dilutions used.

anti-UCP1 (Abcam, ab10983)
anti-GAPDH (14C10) (Cell Signaling Technology, #2118)
Total OXPHOS Rodent WB Antibody Cocktail (20E9DH10C12, 21A11AE7, 13G12AF12BB11, 1D6E1A8, 15H4C4) (Abcam, ab110413)
anti-Tyrosine Hydroxylase (Sigma-Aldrich, AB1542)
anti-RNA Pol II (4H8) (Active Motif, #39097)
anti-SIRT7 (D3K5A) (Cell Signaling Technology, #5360)
anti-IGFBP2(IMP2) (MBL, #RN008P)
anti-DYKDDDDK (FLAG) tag (1E6) (Fujifilm Wako Pure Chemical Corporation, #018-22381)
anti-HA tag (3F10) (Roche Applied Science, #11867423001)
anti-acetylated-lysine (Cell Signaling Technology, #9441)
anti-GAL4 DNA-BD (Sigma–Aldrich, #G3042)
HRP-conjugated Affinipure Goat anti-Rabbit IgG(H+L) (Proteintech, #SA00001-2)
HRP-conjugated Affinipure Goat anti-Mouse IgG(H+L) (Proteintech, #SA00001-1)
HRP-conjugated Affinipure Goat anti-Rat IgG(H+L) (Proteintech, #SA00001-15)
HRP-conjugated Affinipure Rabbit anti-Sheep IgG(H+L) (Proteintech, #SA00001-16)

Validation

All antibodies used in this study were obtained from commercial sources. The details of manufactures' validation can be found on the web site of each manufacture.

anti-UCP1 (Abcam, ab10983)
https://www.abcam.co.jp/ucp1-antibody-ab10983.html
anti-GAPDH (Cell Signaling Technology, #2118)
https://www.cellsignal.jp/products/primary-antibodies/gapdh-14c10-rabbit-mab/2118
Total OXPHOS Rodent WB Antibody Cocktail (Abcam, ab110413)
https://www.abcam.co.jp/total-oxphos-rodent-wb-antibody-cocktail-ab110413.html
anti-Tyrosine Hydroxylase (Sigma-Aldrich, AB1542)
https://www.merckmillipore.com/JP/ja/product/Anti-Tyrosine-Hydroxylase-Antibody,MM_NF-AB1542
anti-RNA Pol II (Active Motif, #39097)
https://www.activemotif.com/catalog/details/39097/rna-pol-ii-antibody-mab
anti-SIRT7 (Cell Signaling Technology, #5360)
https://www.cellsignal.jp/products/primary-antibodies/sirt7-d3k5a-rabbit-mab/5360
anti-IGFBP2(IMP2) (MBL, #RN008P)
https://www.mblbio.com/bio/g/dtl/A/index.html?pcd=RN008P
anti-DYKDDDDK (FLAG) tag (Fujifilm Wako Pure Chemical Corporation, #018-22381)
https://labchem-wako.fujifilm.com/jp/product/detail/W01W0101-2238.html
anti-HA tag (Roche Applied Science, #11867423001)
https://www.sigmaaldrich.com/JP/ja/product/roche/roahaha
anti-acetylated-lysine (Cell Signaling Technology, #9441)
https://www.cellsignal.jp/products/primary-antibodies/acetylated-lysine-antibody/9441
anti-GAL4 DNA-BD (Sigma–Aldrich, #G3042)
https://www.sigmaaldrich.com/JP/ja/product/sigma/g3042
HRP-conjugated Affinipure Goat anti-Rabbit IgG(H+L) (Proteintech, #SA00001-2)
https://www.ptglab.co.jp/products/HRP-conjugated-Affinipure-Goat-Anti-Rabbit-IgG-H-L-secondary-antibody.htm
HRP-conjugated Affinipure Goat anti-Mouse IgG(H+L) (Proteintech, #SA00001-1)
https://www.ptglab.co.jp/products/HRP-conjugated-Affinipure-Goat-Anti-Mouse-IgG-H-L-secondary-antibody.htm
HRP-conjugated Affinipure Goat anti-Rat IgG(H+L) (Proteintech, #SA00001-15)
https://www.ptglab.co.jp/products/HRP-conjugated-Affinipure-Goat-Anti-Rat-IgG-H-L-secondary-antibody.htm
HRP-conjugated Affinipure Rabbit anti-Sheep IgG(H+L) (Proteintech, #SA00001-16)
https://www.ptglab.co.jp/products/Peroxidase-conjugated-Affinipure-Rabbit-Anti-Sheep-IgG-H-L.htm

Eukaryotic cell lines

Policy information about cell lines

Cell line source(s)

HEK293T cells were obtained from Clontech.
293AAV cells were obtained from Cell Biolabs.

Authentication

None of the cell lines were authenticated in this study, because we directly obtained these cell lines from company.

Mycoplasma contamination

The cell line was tested negative for mycoplasma contamination.

Commonly misidentified lines
(See ICLAC register)

None of the used cell lines is listed in ICLAC database.

Animals and other organisms

Policy information about studies involving animals; ARRIVE guidelines recommended for reporting animal research

Laboratory animals

All experimental procedures were approved by the Kumamoto University Ethics Review Committee for Animal Experimentation (Approval ID: A27-024, A29-001, A 2019-048, A 2021-001). All mice were housed at a maximum of 5 mice/cage and maintained in a climate-controlled environment at approximately 22–23°C and 40–80% humidity under specific pathogen-free conditions and strict 12-h light/dark cycles and had access to regular chow (CE-2, CLEA Japan Inc.) and water ad libitum, unless otherwise specified. Our Sirt7 KO mice (ref. 64) and another independent line of Sirt7 KO mice (ref. 37) were back-crossed for over five generations with C57BL/6J (CLEA Japan). Only the heterozygotes were bred (Sirt7+/– × Sirt7+/–), and the littermates (WT and Sirt7 KO mice) were used

for studies. These two lines of Sirt7 KO mice did not exhibit a phenotype with partial embryonic lethality, postnatal death, growth retardation, and a progeroid-like features, as shown by another Sirt7 KO mouse strain (JAX012771). Adipoq-Cre (JAX010803) (ref. 48) and Ucp1-Cre (JAX024670) (ref. 49) mice, which were back-crossed for over eight generations with C57BL/6J, were a kind gift from Dr. Evan D. Rosen and were crossed with Sirt7 floxed mice (Sirt7fl/fl) (ref. 35) for generation of Sirt7 AdKO mice and Sirt7 BAdKO mice, respectively.

Details of number, gender, and age were included in the corresponding figure legends. Summary of mice used here as follows:
15-week-old male WT and Sirt7 KO mice.
10-week-old male WT and Sirt7 KO mice.
11-week-old male WT and Sirt7 KO mice.
16-week-old female WT and Sirt7 KO mice.
2-month-old male WT and another independent line of Sirt7 KO mice (from Dr. Johan Auwerx).
2-year-old male WT and Sirt7 KO mice.
18-week-old male C57BL/6J mice.
13-week-old male WT and Sirt7 KO mice.
20-week-old male WT and Sirt7 KO mice.
1-year-old male WT and Sirt7 KO mice.
12-week-old male Adipoq-Cre control and Sirt7 AdKO mice.
20-week-old male Adipoq-Cre control and Sirt7 AdKO mice.
11-week-old male Adipoq-Cre control and Sirt7 AdKO mice.
10-week-old male Adipoq-Cre control and Sirt7 AdKO mice.
12-week-old male Ucp1-Cre control and Sirt7 BAdKO mice.
20-week-old Ucp1-Cre control and Sirt7 BAdKO mice.

Wild animals

Not used.

Field-collected samples

Not used.

Ethics oversight

All experimental procedures were approved by the Kumamoto University Ethics Review Committee for Animal Experimentation (Approval ID: A27-024, A29-001, A 2019-048, A 2021-001).

Note that full information on the approval of the study protocol must also be provided in the manuscript.
